# Photocatalytic Degradation of Diclofenac by Hydroxyapatite–TiO_2_ Composite Material: Identification of Transformation Products and Assessment of Toxicity

**DOI:** 10.3390/ma11091779

**Published:** 2018-09-19

**Authors:** Sapia Murgolo, Irina S. Moreira, Clara Piccirillo, Paula M. L. Castro, Gianrocco Ventrella, Claudio Cocozza, Giuseppe Mascolo

**Affiliations:** 1 CNR, Istituto di Ricerca Sulle Acque, Via F. De Blasio 5, 70132 Bari, Italy; sapia.murgolo@ba.irsa.cnr.it (S.M.); gianrocco.ventrella@uniba.it (G.V.); 2 CBQF—Centro de Biotecnologia e Química Fina, Laboratório Associado, Escola Superior de Biotecnologia, Universidade Católica Portuguesa/Porto, Rua Arquiteto Lobão Vital, 172, 4200-374 Porto, Portugal; ismoreira@porto.ucp.pt (I.S.M.); plcastro@porto.ucp.pt (P.M.L.C.); 3 CNR, Institute of Nanotechnology, Campus Ecoteckne, Via Monteroni, 73100 Lecce, Italy; clara.piccirillo@nanotec.cnr.it; 4 Dipartimento di Scienze del Suolo, della Pianta e degli Alimenti—Di.S.S.P.A., Università di Bari, Via Amendola 165/A, 70126 Bari, Italy; claudio.cocozza@uniba.it

**Keywords:** diclofenac, hydroxyapatite, photocatalysis, transformation products, toxicity

## Abstract

Diclofenac (DCF) is one of the most detected pharmaceuticals in environmental water matrices and is known to be recalcitrant to conventional wastewater treatment plants. In this study, degradation of DCF was performed in water by photolysis and photocatalysis using a new synthetized photocatalyst based on hydroxyapatite and TiO_2_ (HApTi). A degradation of 95% of the target compound was achieved in 24 h by a photocatalytic treatment employing the HApTi catalyst in comparison to only 60% removal by the photolytic process. The investigation of photo-transformation products was performed by means of UPLC-QTOF/MS/MS, and for 14 detected compounds in samples collected during treatment with HApTi, the chemical structure was proposed. The determination of transformation product (TP) toxicity was performed by using different assays: *Daphnia magna* acute toxicity test, Toxi-ChromoTest, and *Lactuca sativa* and *Solanum lycopersicum* germination inhibition test. Overall, the toxicity of the samples obtained from the photocatalytic experiment with HApTi decreased at the end of the treatment, showing the potential applicability of the catalyst for the removal of diclofenac and the detoxification of water matrices.

## 1. Introduction

The most common wastewater treatment plants (WWTPs) used worldwide are mainly based on the activated sludge technique and are not able to remove and/or degrade several families of trace compounds, known as “contaminants of emerging concern” (CECs). Particularly, the use of pharmaceuticals (PhACs) in everyday human life, continually introduced from the market for the healthy population and easily accessible without a prescription, represents a relevant source of contamination of the aquatic environment, as several of these are not completely metabolized by the human organism; they are, therefore, eliminated by urine and faeces [1,2,3].

Moreover, biological processes in WWTPs as well as subsequent chemical treatments may convert PhACs into transformation products (TPs) that can be more toxic than their parent compounds [4,5,6]. Several toxicological and ecotoxicological studies have been performed to obtain an overview of the risk assessment of TPs detected in waters and wastewaters [7,8,9].

Both trace compounds and their relative TPs have been found in different environmental compartments, including WWTP effluent, surface water, groundwater, drinking water, soil, sediments, and sludge, and their concentration ranged from μg L^−1^ to ng L^−1^. At present, CECs are not regulated, but the relevance of addressing this issue was acknowledged by the Directive 2013/39/EU listing priority substances and further supported by the implementation of Decision (EU) 2015/495 [10]. In addition, no relevant knowledge is available about the potential ecological impacts of CECs. The continuous discharge of and long-term exposure to these contaminants, however, may have long-term effects on human health. Reproductive impairment, hormonal dysfunction, development of antibiotic-resistant bacteria, and cancer may be possible consequences [11,12].

Among the PhACs, biodegradation of nonsteroidal anti-infiammatory drugs (NSAIDs) has been found to be slow or negligible. Several reviews published in recent years have discussed the occurrence, persistence, and fate of the diclofenac (DCF). DCF is one of the most detected PhACs in the water matrix and it is recalcitrant to conventional wastewater treatment plants. Data reported in the literature have shown that WWTPs remove DCF with an efficiency in the range of 21–40% [13,14,15,16].

After consumption, DCF is mostly transformed into hydroxyl metabolites from human metabolism and subsequently excreted from the body in the aqueous environment. Furthermore, more DCF metabolites are present in aqueous matrices due to biological processes in WWTPs which convert DCF to hydroxydiclofenac; the same process takes place through a photochemical process, i.e., photolysis. These data highlight that not only DCF but also its metabolites and photo-transformation products with different physicochemical properties can be sources of contamination in the aqueous environment [17,18].

The literature reports that the combination of biological processes with advanced oxidation processes (AOPs) led to increased efficiency in the removal of organics [19,20]; because of this, a great amount of work has been published on AOPs as efficient treatments for the removal of PhACs and their metabolites in water [21,22,23]. Among the AOPs, particular attention has been given to heterogeneous photocatalysis based on materials such as TiO_2_, as they represent a promising and environmentally sustainable wastewater treatment technology. Their activity is based on the formation of highly reactive OH radicals which are nonselective and have a high oxidative power (E0 = +2.80 V) [24,25]. In the field of photocatalysis, a large number of new TiO_2_-based photocatalysts have been developed combining TiO_2_ with other materials that can enhance its performance [26,27,28,29]. The combination with hydroxyapatite (Ca_10_(PO_4_)(OH)_2_, HAp), in particular, seems very promising due to its excellent absorption properties. In fact, in a multiphasic HAp-TiO_2_ material, pollutants get adsorbed on the surface more easily, leading to a higher degradation rate ([30] and references therein).

A recent study showed that it is possible to obtain a photocatalyst based on HAp and TiO_2_ using cod fish bones as a source; HAp, in fact, is the main component in human and animal bones. Piccirillo et al. showed that treating the bones in a Ti (IV)-containing solution and successively calcining them leads to a photoactive multiphasic material [31]. This material was tested in the degradation of DCF in aqueous matrices using a bench-scale system [32]. Results showed that when compared to photolysis, HAp-titania significantly increases the diclofenac removal and is also effective when employed in a complex matrix (wastewater) or when reused in subsequent cycles of treatments. FTIR measurements of the photocatalyst taken before and after the photodegradation experiments showed that the powder material did not present any significant property change [32].

However, measurements of total organic carbon at the end of the experiments showed incomplete mineralization of the target pollutant. This indicates the production of some TPs which can be less biodegradable and/or more toxic than the parent compound.

Previous studies have investigated diclofenac TPs in water matrices after photocatalytic degradation [33,34,35]; to the best of our knowledge, however, no work has been done when hydroxyapatite-based catalysts were used.

The aim of this work is, therefore, a follow-up of the experimental results described in E. Màrquez Brazòn et al.’s work [32] in order to investigate the possible presence of photo-transformation products of diclofenac in water after photolysis and photocatalysis with hydroxyapatite and TiO_2_ (HApTi) and to assess their level of toxicity in comparison with the parent DCF.

The investigation of TP structure was performed by means of an analytical approach based on high-resolution mass spectrometry coupled with liquid chromatography (LC-HRMS), providing a proposed molecular formula and elucidating the respective chemical structures [36]. Considering the proposed chemical structures of the identified TPs, the degradation mechanism of DCF with only photolysis and with photocatalysis (in the presence of a hydroxyapatite-based catalyst) was compared. Finally, for a complete risk valuation study on TPs of DCF formed during the investigated treatments, determination of their toxicity was performed by using different assays: *Daphnia magna* acute toxicity test, Toxi-ChromoTest, and *Lactuca sativa* and *Solanum lycopersicum* germination inhibition test.

## 2. Materials and Methods

### 2.1. Chemicals

DCF was purchased from Sigma-Aldrich (Steinheim, Germany). HPLC-grade methanol (Riedel-de Haën, Baker) was used to prepare a stock standard solution of DCF as well as for ultra-performance liquid chromatography (UPLC) analysis. Ultrapure water (18.2 MΩcm, organic carbon ≤ 4 μg/L) was supplied by a Milli-Q water system (Gradient A-10, Millipore SAS, Molsheim, France) and used for both UPLC analysis and to perform investigated treatments. All the analysed samples were previously filtered on syringe CA membrane filters with a 0.45-µm pore size.

### 2.2. Synthesis and Characterisation of HApTi

A detailed description of the photocatalytic material preparation and characterization was previously published [31]. Briefly, the photocatalyst was prepared from cod fish bones, which were treated in a basic Ti(SO_4_)_2_ solution and successively calcined at 800 °C. The powder was constituted by hydroxyapatite (HAp), tricalcium phosphate, and TiO_2_ (anatase) in a proportion of 54, 45, and 1 wt %, respectively. For a more complete description of the material, see the reference literature.

### 2.3. Bench-Scale Experiments

Photocatalysis experiments were performed as previously described [32]. Briefly, a volume of 50 mL of DCF solution at 5 mg/L in distilled water was placed in 100-mL flasks with 0.2 g of photocatalyst to achieve a concentration of 4 g/L. The flasks were magnetically stirred and irradiated from the top with a XX-15 BLB UV lamp (λ 365 nm) (see reference [37] for the full emission spectrum); the irradiation density was 1.80 mW/cm^2^ (value given by the producer). Experiments were also performed as described above but with no photocatalyst to monitor the DCF degradation due to UV illumination. Before starting the experiments, the solutions were left stirring in the dark for 30 min to ensure that removal of DCF was due to photodegradation and not to adsorption on the surface of the photocatalyst. Samples were taken at regular intervals to assess the degradation of DCF. DCF concentration was determined by a validated HPLC-UV method [38,39]. Experiments were performed in duplicate. New experiments were performed in triplicate for the identification of the TPs. Samples were analysed by UPLC-QTOF/MS/MS as described in the following section.

### 2.4. Analytical Setup and Data Processing

An Ultimate 3000 System (Thermo Fisher Scientific, Waltham, MA, USA) interfaced with a TripleTOF 5600+ high-resolution mass spectrometer (AB-Sciex), equipped with a duo-spray ion source operated in electrospray (ESI) mode (in positive and negative ion modes), was used to identify TPs. An information-dependent acquisition (IDA) method was used.

The mass spectrometer was calibrated using standards recommended by AB SCIEX for calibrating the AB SCIEX TripleTOF^®^ 5600 Instrument, i.e., ESI Positive Calibration Solution and ESI Negative Calibration Solution. These solutions consist of a list of TOF MS Calibration Ions in the range of 140–1500 Da, including reserpine and sulfinpyrazone for MS/MS calibration in positive and negative mode, respectively, for which the list of product ions is known. All the information related to these lists are saved in reference tables available in the calibration section of the instrument. Briefly, before a run of analyses, a manual calibration was carried out by injecting the calibration solution by means of a Calibrant Delivery System (CDS) in tuning mode. Each calibration solution was configured on the CDS by a specific valve position. The instrument was therefore calibrated by comparing the exact mass of the acquired ions (experimental mass) with respect to that of the ions listed in the reference tables (theoretical mass), both in TOF MS and Product Ion MS/MS mode. When the batch was submitted, the calibration samples were inserted into the queue every five samples. Each run started with a calibration sample. With the CDS configured, the software automatically created a calibration method that matched the acquisition method that was used for the next sample in the queue. Calibration data were saved to a separate data file for each calibration sample.

The MS interface conditions for sample acquisition were the following: curtain gas (psig) 35, ion source gas 1 nebulizer gas (psig) 55, ion source gas 2 turbo gas (psig) 55, IonSpray voltage (V) 5500 (positive mode) and −4500 (negative mode), source temperature (°C) 450, declustering potential (V) 80, *m*/*z* range 100–1000 Da.

A Waters BEH C18 column 2.1 × 150 mm, 1.7 µm, was used for the chromatographic separation, operating at a flow of 0.200 mL/min. Five-microliter samples were injected and eluted with a binary gradient consisting of 1.5 mM ammonium acetate in H_2_O/MeOH 95/5 (A) and 1.5 mM ammonium acetate in methanol (B) as follows: 0% B at the initial point, linearly increased to 95% in 12 min, and held for 5 min. A 7-min equilibration step at 0% B was used at the end of each run to bring the total run time per sample to 24 min. As for the data processing, both suspect target and nontarget screenings, with identification of already known in the literature and novel TPs, respectively, were carried out. Specifically, all the collected samples corresponding to the reaction times for a specific treatment were processed by a specific analytical protocol, including two steps:

- Suspect screening: The AB-Sciex software, i.e., SciexOS 1.2, PeakView 2.2, MasterView 1.1, and LibraryView 1.1.0, were employed by using a list of likely TPs collected from the literature or from prediction models. The samples were screened for those candidates on the basis of the mass exact, isotopic pattern, fragmentation MS/MS patter, and chromatographic retention time. However, since no reference standards are available for all revealed TPs, the subsequent confirmation of the analytes is not completely possible. Therefore, the molecular formula and structure of suspected molecules can be only predicted.
- Nontarget screening: An open source software, i.e., enviMass 3.5 [40], was used for the investigation of compounds for which no previous knowledge is available and which is usually carried out after suspect screening. Briefly, after a first step of peak picking, the following steps include the removal of peaks found also in the blank sample, the mass recalibration, and the componentization of isotopes and adducts.

The assignment of the molecular formula to the accurate mass of the selected peak was performed using AB-Sciex software. Moreover, based on the interpretation of the fragmentation pattern generated in MS/MS acquisition, a candidate structure was assigned to the identified TPs, which provided a tool to hypothesize a possible degradation pathway.

### 2.5. Toxicity Test

The toxicity of DCF solutions was evaluated through bioassays in samples collected at the times 0, 4, 6, and 24 h in the duplicate experiments, initially performed both with and without photocatalyst for assessing the DCF decay. Samples were analysed without dilution in order to assess the evolution of whole sample toxicity during photodegradation experiments and to compare the resulted final toxicity in the presence and absence of the photocatalytic material. All toxicity assays were performed in quadruplicate of combined duplicate samples from photodegradation experiments.

The 24–48-h immobilization of crustaceans *D. magna* bioassays were performed using Daphtoxkit F Magna (MicroBio Tests, Mariakerke (Gent), Belgium) following manufacturer recommendations. The toxicity was measured as the immobilization of *D. magna* according to the procedures of OCED Guideline 202 [41] and ISO 6341 [42] after 15-min exposure.

The Toxi-ChromoTest (EBPI, Mississauga, ON, Canada) was used according to manufacturer instructions to determine the samples’ potential for the inhibition of the de novo synthesis of an inducible β-galactosidase by an *E. coli* mutant. The activity of the enzyme was detected by the hydrolysis of a chromogenic substrate.

The acute bioassay with *L. sativa* and *S. lycopersicum* evaluated the potential toxicity considering the inhibition of seed germination and root and shoot elongation according to OECD Guideline 208 [43]. Experiments were performed in triplicate at 25 °C for 7 days. The inhibition normalized on negative control data were expressed as percentage of effect.

## 3. Results and Discussion

**Figure 1:**
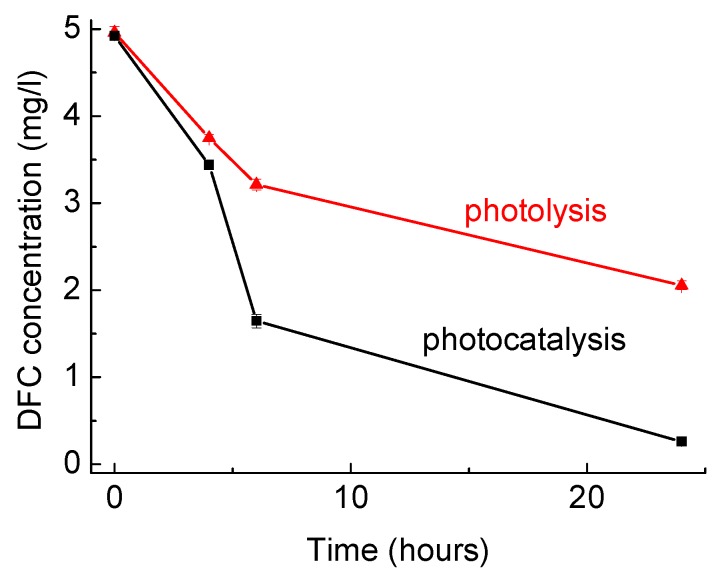
Diclofenac (DCF) photodegradation over time by UV photolysis (no photocatalyst) and UV photocatalysis employing hydroxyapatite and TiO_2_ (HApTi) catalyst in solution (DCF concentration 5 ppm; HApTi catalyst concentration 4 g/L). Error bars refer to standard deviation of duplicate samples.

In the present work, the experimental setup used for photodegradation was slightly changed compared to previous experiments [32] to have a higher irradiation dose and, hence, higher degradation efficiency. The results of photodegradation of DCF plotted in Figure 1 showed that in the photolytic assay, i.e., containing no catalyst, a removal of about 60% of the target compound was observed, and the presence of the HApTi catalyst increased the degradation to 95% at the end of the experiment, demonstrating the photocatalytic efficiency of the catalyst for DCF removal.

### 3.1. Identification of the Transformation Products Produced by DCF Photodegradation

Although almost 95% and 60% of DCF was removed under the investigated treatment in the presence of HApTi and only UV light, respectively. The TOC measurements showed that the pharmaceutical was not completely mineralized [32]. From this perspective, samples collected at different irradiation times (0, 4, 6, and 24 h) during both photolytic and photocatalytic experiments were analysed for TPs by UPLC/ESI-QTOF-MS-MS. The acquired chromatograms showed several peaks, of which only the main ones were identified by merging output results of both the suspect and nontarget screening protocol. In the first step of identification, the samples were processed by querying the detected peaks with a list of the main TPs collected from already published works in which DCF photodegradation was investigated [15,33,44,45,46,47]. Based on the analyses acquired in full scan mode as well as on the corresponding exact masses, considering both the isotopic cluster of molecular ions and the fragmentation pattern, 10 major photo-transformation products were identified. The analytical protocol employed for nontarget screening also allowed the detection of four new photo-transformation products clearly showing in the mass spectra the characteristic isotopic cluster of chlorine-containing compounds.

**Figure 2:**
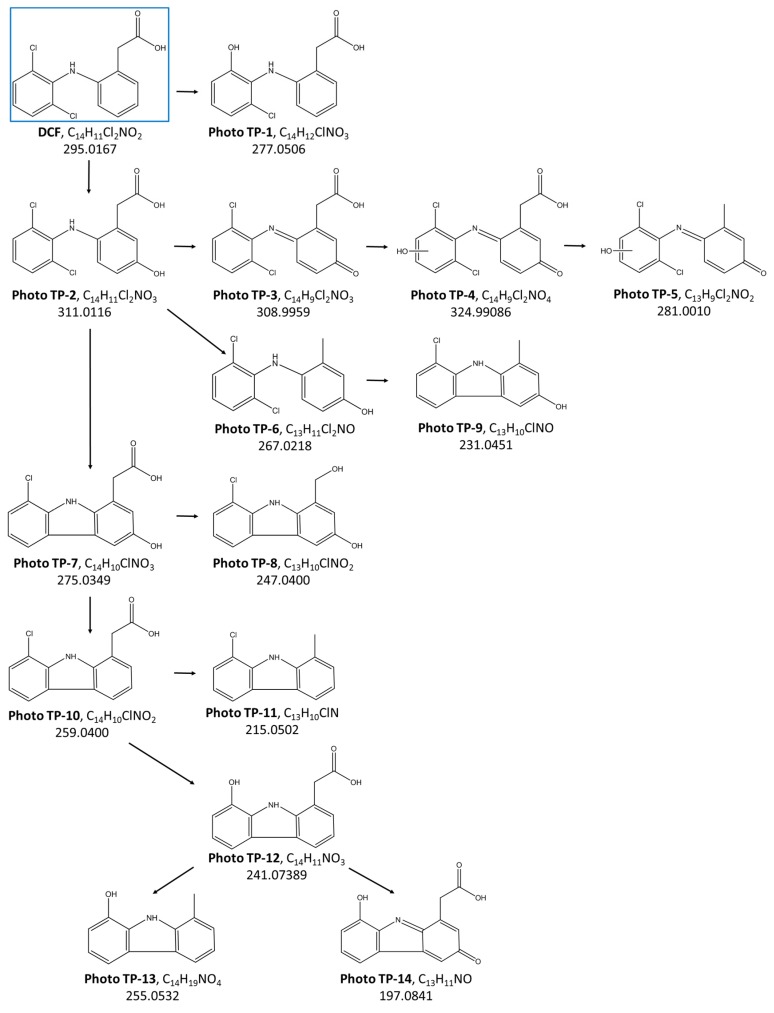
DCF degradation pathway proposed for the Photo TPs identified during treatment with HApTi.

In Table 1, the accurate masses (calculated and measured) of the 14 detected Photo TPs, the ionization mode acquisition, the predicted formula, and the calculated mass errors are listed. The accurate mass results were found with an error in a range between −2 and +1 ppm, thus providing the assignment of proposed elemental compositions with confidence. For each potential photo-transformation product, the elucidation of the structure was assessed based on the interpretation of the accurate mass of the MS/MS fragments as well as considering all the reaction mechanisms likely to occur in the oxidation processes applied for DCF removal, i.e., photocatalysis using HApTi as a catalyst (Figure 2). Oxidation is one of the major degradation mechanisms during photocatalytic treatment. Indeed, the formation of oxidized compounds was confirmed by the identification of Photo TPs, the predicted formula of which showed an increase of oxygen atoms with respect to the parent DCF elemental composition. An oxidative displacement of chloride from DCF was proposed for Photo TP-1, with a decrease of 18 Da in terms of *m*/*z* resulting from the loss of HCl and subsequent hydroxylation, whereas an increase of 16 Da was indicative of DCF hydroxylation in Photo TP-2. Although MS data were not enough to define the exact position of the hydroxyl group, the fragment *m*/*z* 166.0643 detected on Photo TP-2 (Table 1) proved that the hydroxylation occurred on the nonchlorinated ring.

Photo TP-3 revealed two fewer hydrogen atoms with respect to Photo TP-2 with the formation of an additional double bound. The presence of the carboxylic acid functionality confirmed by the MS/MS fragment (fragment showed the loss of CO_2_) led to assigning the structure of a benzoquinone imine species to the Photo TP-3. For Photo TP-4, a mass increase of 16 Da in relation to Photo TP-3 reveals a subsequent addition of one oxygen atom; according with the fragmentation pattern, this could be explain by a hydroxylation on the chlorinated ring. The Photo TP-5 (MW 281 Da) and Photo TP-6 (MW 267 Da) were rationalized to be formed from Photo TP-4 and Photo TP-2, respectively, both by a decarboxylation reaction of the aliphatic chain.

**Figure 3:**
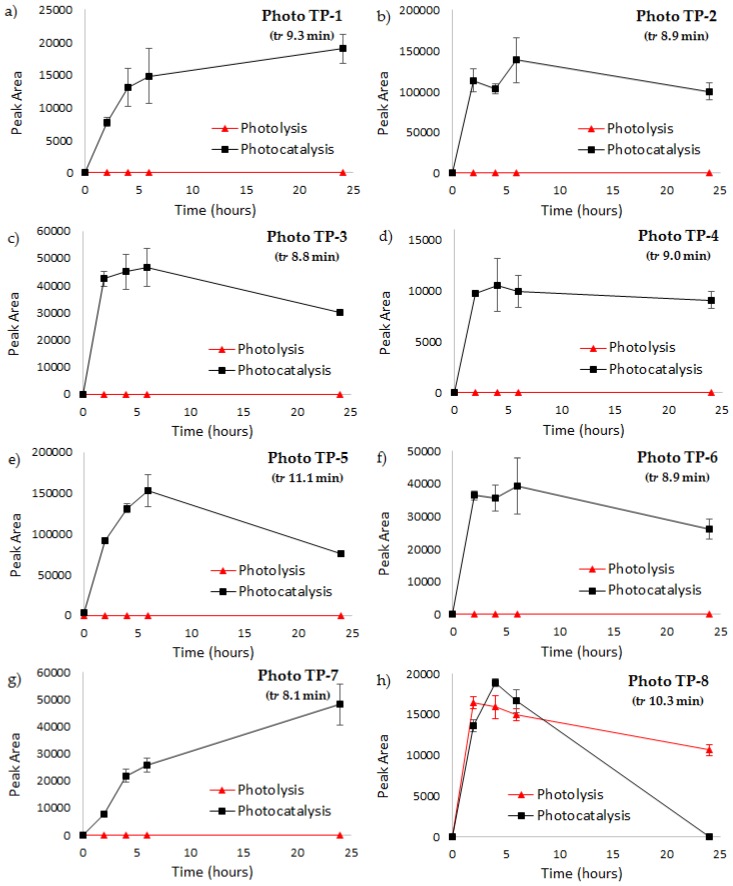

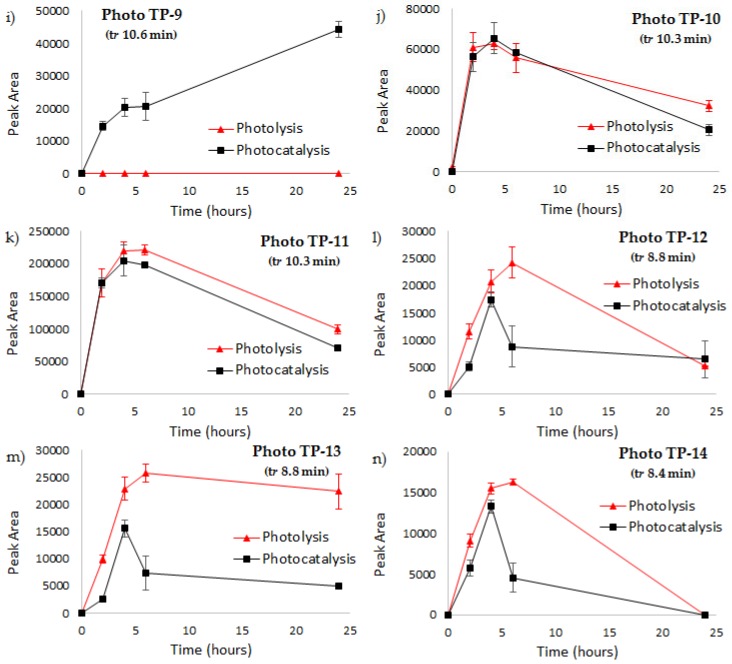
Time profiles of detected Photo TPs during investigated treatments, namely, photolysis (only UV light) and photocatalysis (UV light in the presence of HApTi). Error bars refer to standard deviation of triplicate samples.

From plotting the time profiles of the identified Photo TPs (Figure 3), it is evident that Photo TP-1, TP-2, TP-3, TP-4, TP-5, and TP-6 are formed only during the photocatalytic treatment in the presence of HApTi, with an intensity that significantly increased during the first hours of treatment (Figure 3a–f).

The loss of one chlorine atom from Photo TP-1 and the subsequent cyclization reaction, forming a five-membered ring with nitrogen, generated Photo TP-7. The time profiles show that TP-7 formation increases with the time of photocatalytic treatment, as does Photo TP-9, which forms from Photo TP-6 by a loss of one chlorine atom (Figure 3g,i). Photo TP-8 is formed both during photolysis and photocatalysis, but only the latter treatment was able to quickly remove it (Figure 3h).

As for Photo TP-10, the isotopic pattern of which indicates the presence of only one chlorine atom, a chlorocarbazole acetic acid structure was proposed. Considering the mass decrease of 44 Da between Photo TP-10 and Photo TP-11 and the predicted formula, Photo TP-11 corresponds to the loss of the carboxylic acid group. Photo TP-12 revealed a mass decrease of 18 Da with respect to Photo TP10; the absence of the typical isotopic pattern of chlorine and the assigned formula for the detected compound could be attributed to the replacement of the chloride atom with the OH group.

The time profiles of these products (Figure 3j–l) reveal no significant difference between photolysis and photocatalysis. In both treatments, the detected TPs achieve the maximum of formation after 4 h of treatment with a subsequent decay. Although the removal is not complete at the end of the treatments, it was slightly faster in the presence of HApTi.

Two more products of MW 255 and 197 Da were observed and the molecular ion isotopic cluster indicated that no chlorine atom was present in both of these products, Photo TP-13, and Photo TP-14, respectively. The photolytic treatment generates Photo TP-13 with higher intensity with respect to photocatalytic treatment, with a subsequently slower removal of the compound over time (Figure 3m), whereas a complete removal for Photo TP-14 at the end of both treatments was observed.

The time profiles of the identified Photo TPs (Figure 3) show the average values of the peak area measured during three subsequent cycles of both photolytic and photocatalytic treatments for DCF removal. In each cycle of photocatalysis, the HApTi catalyst was removed from the aqueous solution by means of a centrifugation step at the end of the treatment and then reused for a new subsequent batch of photocatalytic treatment. The results confirm the general trend of identified Photo TPs, for which some transformation products tend to accumulate in the water matrix and some others are transiently formed with a tendency to disappear. Moreover, the results showed that the photocatalytic activity of HApTi catalyst is approximately the same in different subsequent cycles of treatment.

### 3.2. DCF Degradation: Photolysis versus Photocatalysis with HApTi

**Figure 4:**
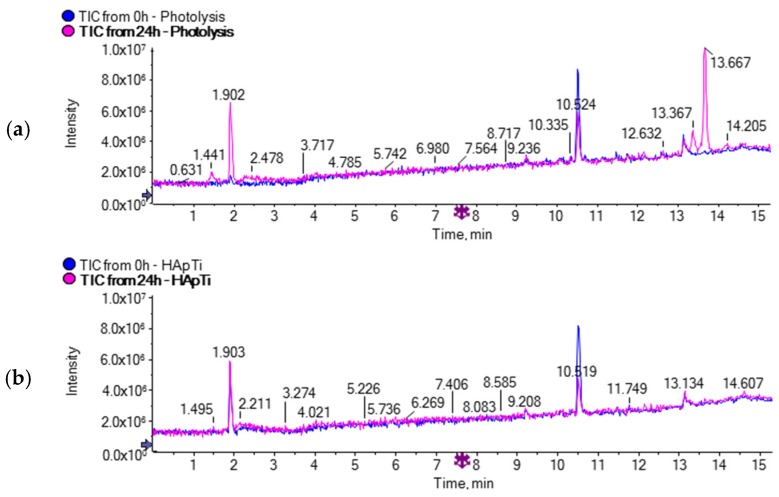
Total ion chromatogram acquired for both (**a**) photolysis and (**b**) photocatalysis in the presence of HApTi comparing time zero with respect to time 24 h.

Based on the 14 identified Photo TPs, a pathway of DCF degradation in the presence of HApTi was proposed (Figure 2). The principal degradation mechanisms were explained by hydroxylation, oxidation, and decarboxylation reactions. Among these products, six Photo TPs were also identified during photolytic treatment (Figure 3 h, j–n) in the presence of only UV light. Moreover, 22 additional molecular ions (Table 2) showing significant abundance only in the LC-MS chromatograms of photolysis samples by comparing the initial and the last sample (time zero with respect to reaction time 24 h) were detected between 13.3 and 13.6 min (Figure 4) by nontarget screening in positive mode ionization.

An accurate processing of the 20 detected molecular ions by AB-Sciex software allowed us to define for each one a consistent elemental composition based on the exact mass and isotopic pattern. The obtained elemental compositions seem to be mainly nitrogen-containing compounds, but since not enough data are available in the literature about these products, further investigation would be needed in order to identify a relative molecular structure.

These 20 TPs were detected only in samples collected during photolytic treatment; indeed, no evidence of such TPs was found in samples subjected to photocatalytic treatment in the presence of HApTi. This clearly indicates that the photodegradation mechanisms are significantly different when the HAp-based photocatalyst is employed.

It is known that different reactive oxygen species (ROS) are formed with a photocatalyst under light irradiation [30], including •OH, O_2−_, and h+. Depending on the nature of the catalyst and the experimental conditions, some ROS will prevail over others and will determine the photodegradation mechanism [48]. This affects the nature of the TPs formed during degradation. Regarding the HApTi photocatalyst, some studies report •OH as the main species [49,50]. The present investigation seems to confirm these data, as the identified TPs PhotoTP-1 and TP-2 both have an OH group added to the parent compound. Neither of these products was detected in photolysis experiments (see Figure 3a,b); this confirms a different mechanism that does not involve such ROS.

The TPs identified for the experiment without the catalyst (Figure 3) seem to indicate that UV irradiation induces the breaking of some chemical bonds (i.e., C–Cl) and the formation of some others (i.e., N–H) in the DCF molecule. The identification of the TPs listed in Table 2 could give some further clarifications. This study will be performed in the future as a separate investigation; no further details were added here since the main aim of the present work was to assess the performance of the HApTi photocatalyst in terms of TP toxicity.

### 3.3. Evaluation of Toxicity of the Treated Water

**Figure 5:**
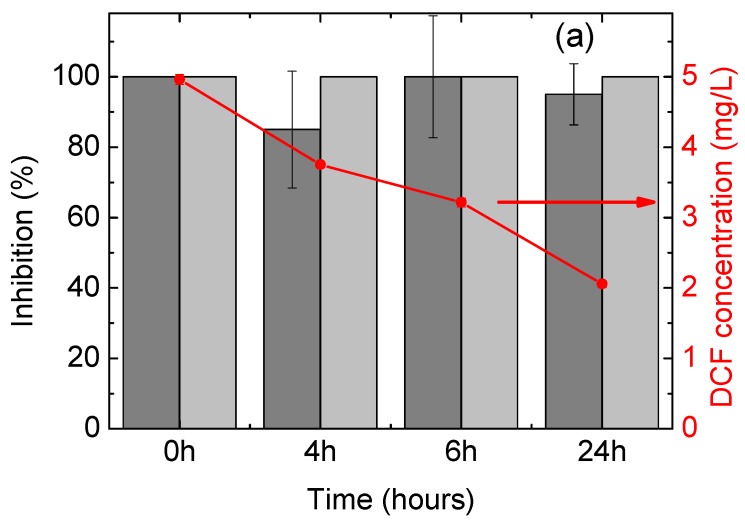

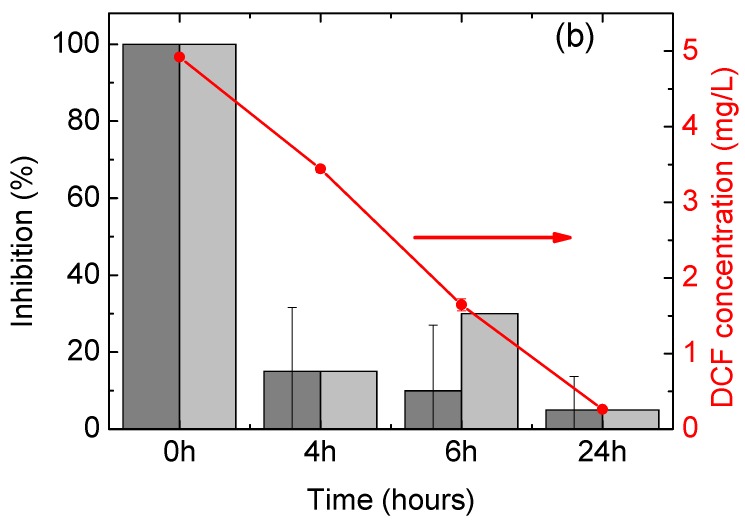
Acute toxicity on *Daphnia magna* during photodegradation of DCF using (**a**) UV light and (**b**) UV light + HApTi. Bars in dark grey refer to 24-h immobilization, while those in pale grey to 48-h immobilization (values to be read on the left axis). Error bars refer to standard deviation of quadruplicate measurements of combined duplicate samples. DCF concentration is reported on the right axis and error bars refer to standard deviation of duplicate samples.

Figure 5 shows the evolution of the acute toxicity on *D. magna* during photodegradation experiments. DCF solutions (5 mg/L) at the beginning of the experiments exhibit high toxicity on *D. magna* (100% inhibition). It can be seen that the toxicity of the water samples obtained from the photolysis experiment (i.e., no catalyst, Figure 5a) did not vary significantly and remained very high after 24 h of UV irradiation; similar results were observed for a 48-h exposition. When the photocatalyst was employed (Figure 5b), however, the toxicity drastically decreased after 4 h (15% inhibition) and completely disappeared at the end of the experiment (24 h).

These results indicate that, despite DCF mineralization not being complete, a significant reduction of water toxicity was achieved; this makes photodegradation with the HApTi catalyst a very suitable method for water decontamination.

**Figure 6:**
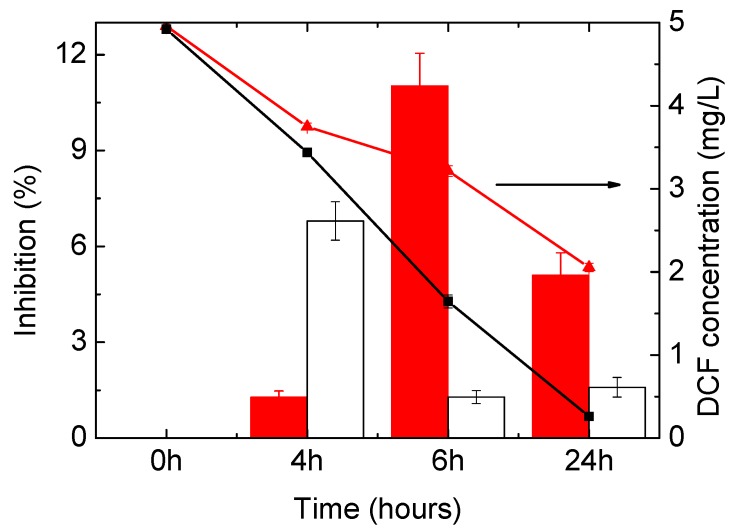
Acute toxicity on mutant bacteria using the Toxi-ChromoTest kit. Red and white bars refer to photolysis and photocatalysis experiments, respectively; the values should be read on the left axis. DCF concentration is reported for both experiments on the right axis—red and black curves respectively. Error bars refer to standard deviation of quadruplicate measurements of combined duplicate samples.

As opposed to what happened in the test with *D. magna*, in the Toxi-ChromoTest, the original DCF solution did not present any toxicity to the mutant bacteria, which was able to produce the β-galactosidase enzyme and convert the chromogenic substrate at levels similar to negative control (Figure 6). In the photolysis experiment, the toxicity factor increased till 6 h, reaching a maximum of 11% inhibition; it successively decreased. In the presence of the catalyst, the toxicity increased faster, reaching its maximum at 4 h (6.8%) but then decreased, with the final toxicity being lower than that observed in the experiment in the absence of the catalyst. Since the original solution of DCF did not show toxicity, it can be concluded that the toxicity observed in this test for the samples from the photodegradation experiments is exclusively due to the toxicity of the products formed. The difference in toxicity between the two experiments can be related to the different rates at which the TPs are formed and then degraded; such a rate is faster when the HApTi photocatalyst is employed.

**Figure 7:**
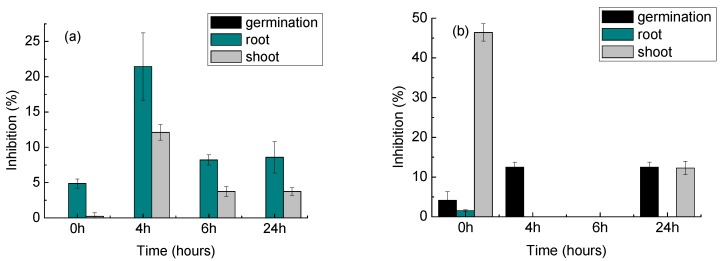
Acute toxicity on (**a**) *Lactuca sativa* and (**b**) *Solanum lycopersicum* during photodegradation of DCF using UV light only (i.e., no catalyst). Experiments with the catalyst showed no toxicity at all (data not shown). Error bars refer to standard deviation of quadruplicate measurements of combined duplicate samples.

The toxicity trend of *L. sativa* and *S. lycopersicum* exposed to DCF during photodegradation sample (no catalyst) is reported in Figure 7; data for the experiments with the catalyst are not shown since no inhibition at all was observed for the whole photocatalysis process for both seeds. For *L. sativa* (Figure 7a), there was no inhibition of germination for the whole 24-h period of the photolysis experiment. Considering the growth of the germinated seeds, however, a small inhibition of the root growth was observed for the initial DCF sample (0 h). This low toxicity is in agreement with results previously reported for the phytotoxicity of this pharmaceutical on *L. sativa* [1]. The inhibition on root growth increased at 4 h of photolysis; for this time, an inhibition of shoot growth was also registered. The root growth inhibition was lower at 6 and 24 h; however, values still higher than those of the original DCF sample were seen. This is in agreement with literature, since increased phytotoxicity of DCF photolysis transformation products in relation to parent compound was also observed after exposure to sunlight [2]. Moreover, some inhibition was also observed for the shoot growth, which was not registered for the original DCF.

In the case of *S. lycopersicum*, an inhibition of all the analysed parameters was seen with the exposure to the initial DCF sample; also, the inhibition of germination increased during photolysis. This indicates that exposure to DCF intermediates produced in the absence of photocatalyst was more inhibitory for seed development than the initial DCF solution.

These data show that for both seeds, the intermediates produced by photolysis, i.e., with no photocatalyst, are more toxic than the parent DCF compound; for the photocatalytic experiments, on the other hand, no toxic effects were observed. These data show that photocatalysis using the HApTi catalyst is a more effective way to degrade DCF without generating toxic compounds. Faster DCF removal and faster metabolite transformation may also play a role.

## 4. Conclusions

The present investigation demonstrated the effectiveness of the novel multiphasic hydroxyapatite–TiO_2_ material for the photocatalytic treatment of diclofenac. Specifically, the novel material gave rise to same transformation products of the photolytic treatment, but the abundance of most of them was minimized at shorter reaction times. It is worth noting that the novel employed catalyst has a size mesh much greater than a conventional TiO_2_ catalyst and, consequently, its removal at the end of the water treatment process is much easier.

As for the toxicity investigation, it was observed that reduced toxicity of the samples resulted from the photocatalytic experiment in comparison with photolysis and in relation to the original DCF sample. Indeed, the hydroxyapatite–TiO_2_ material was effective at detoxifying the samples containing DCF and DCF transformation products. It led to a significant decrease in the toxicity of the water samples despite DCF mineralization not being complete. Therefore, such a catalyst represents an interesting and eco-safe technology for DCF degradation.

## Figures and Tables

**Table 1:** DCF photo-transformation products detected by UPLC/ESI–QTOF–MS IDA.

Compounds	Ionization Mode	Calculated *m*/*z*	Measured *m*/*z*	ppm Error	Products MS/MS	Predicted Formula	Ref.
Photo TP-1	ESI (+)	278.0579	278.0577	−0.6	168.0794, 196.0755, 232.0508, 260.0470	C14H12ClNO3	[44]
Photo TP-2	ESI (+)	312.0189	312.0186	−1.0	166.0643, 194.0612, 230.0357, 265.9974	C14H11Cl2NO3	[44,47]
Photo TP-3	ESI (+)	310.0032	310.0031	−0.4	166.0657, 201.0345, 263.9987, 291.9941	C14H9Cl2NO3	[44,47]
Photo TP-4	ESI (−)	323.9836	323.9835	−0.3	152.0507, 208.0423, 252.0300, 280.0045	C14H9Cl2NO4	[15]
Photo TP-5	ESI (+)	282.0083	282.0084	0.4	166.0646, 194.0598, 229.0285, 263.9979	C13H9Cl2NO2	-
Photo TP-6	ESI (−)	266.0145	266.0147	0.8	127.0543, 166.0662, 184.0961, 206.0185	C13H11Cl2NO	[44,45]
Photo TP-7	ESI (+)	276.0422	276.0420	−0.8	166.0650, 194.0597, 202.0424, 230.0360	C14H10ClNO3	-
Photo TP-8	ESI (−)	246.0327	246.0329	0.7	141.0214, 164.0516, 200.0265, 228.0234	C13H10ClNO2	-
Photo TP-9	ESI (−)	230.0378	230.0378	−0.1	143.113, 166.0646, 194.0606, 215.0134	C13H10ClNO	-
Photo TP-10	ESI (+)	260.0473	260.0468	−1.8	125.0442, 151.0545, 165.0902, 179.0732	C14H10ClNO2	[33,45]
Photo TP-11	ESI (−)	214.0430	214.0431	1.1	65.9613, 138.0405, 142.9975, 178.0652	C13H10ClN	[45]
Photo TP-12	ESI (−)	240.0666	240.0667	0.3	99.9485, 142.0667, 168.0815, 196.0768	C14H11NO3	[33,45,46]
Photo TP-13	ESI (+)	256.0604	256.0604	−0.1	95.0885, 127.0543, 182.0595, 210.0552	C14H9NO4	[15]
Photo TP-14	ESI (−)	196.0768	196.0768	0.2	59.0159, 135.0128, 152.0321, 168.0804	C13H11NO	[45]

**Table 2:** DCF photo-transformation products detected in positive ionization mode in the 24-h photolysis sample.

Compounds	Measured *m*/*z*	Retention Time (min)	MS Error (ppm)	Predicted Formula	Formula Finder Score	Profile as Function of Time
Ion-1	230.2471	13.37	1	C14H31NO	39.7	Increase
Ion-2	258.2789	14.18	−1	C16H35NO	40	Increase
Ion-3	352.3052	13.66	0.2	C14H37N7O3	18.1	Increase
Ion-4	379.3046	13.67	0	C21H38N4O2	43.1	Increase
Ion-5	383.2867	13.67	0.1	C17H34N8O2	73.7	Increase
Ion-6	396.3316	13.67	−0.8	C16H41N7O4	25.4	Increase
Ion-7	427.3124	13.66	0.4	C19H38N8O3	67.8	Increase
Ion-8	440.3575	13.67	−0.7	C23H45N5O3	23.2	Increase
Ion-9	449.3258	13.66	−0.4	C23H40N6O3	26.3	Increase
Ion-10	459.4875	13.38	0.2	No formula found	0	Increase
Ion-11	471.3387	13.65	−0.1	C21H42N8O4	33.8	Increase
Ion-12	484.3839	13.68	-1	C25H49N5O4	25.6	Increase
Ion-13	493.352	13.65	−0.6	C22H48N6O4S	72.9	Increase
Ion-14	537.3772	13.64	0.7	C20H48N12O3S	40.5	Increase
Ion-15	559.3899	13.64	1	C21H46N14O4	45.3	Increase
Ion-16	572.4355	13.68	−0.2	C26H49N15	37.8	Increase
Ion-17	577.3903	13.69	0.5	C38H48N4O	91.8	Increase
Ion-18	581.4031	13.63	0.7	C25H48N12O4	14.9	Increase
Ion-19	621.4163	13.68	0.3	C30H48N14O	30.6	Increase
Ion-20	704.5142	13.67	−0.6	No formula found	0	Increase
Ion-21	748.5391	13.67	0.1	No formula found	0	Increase
Ion-22	792.5668	13.66	−0.4	No formula found	0	Increase
